# Ultraviolet Radiation Protective and Anti-Inflammatory Effects of *Kaempferia galanga* L. Rhizome Oil and Microemulsion: Formulation, Characterization, and Hydrogel Preparation

**DOI:** 10.3390/gels8100639

**Published:** 2022-10-09

**Authors:** Chuda Chittasupho, Sakdanai Ditsri, Sudarshan Singh, Mayuree Kanlayavattanakul, Natthachai Duangnin, Warintorn Ruksiriwanich, Sirivan Athikomkulchai

**Affiliations:** 1 Department of Pharmaceutical Sciences, Faculty of Pharmacy, Chiang Mai University, Chiang Mai 50200, Thailand; 2 Cluster of Research and Development of Pharmaceutical and Natural Products Innovation for Human or Animal, Chiang Mai University, Chiang Mai 50200, Thailand; 3 Department of Pharmacognosy, Faculty of Pharmacy, Srinakharinwirot University, Nakhonnayok 26120, Thailand; 4 School of Cosmetic Science, Mae Fah Luang University, Chiang Rai 57100, Thailand; 5 Regional Medical Sciences Center 1, Chiang Mai 50180, Thailand

**Keywords:** essential oil, ethyl cinnamate, nitric oxide, sunscreen, Zingiberaceae

## Abstract

Long-term UV radiation exposure can induce skin disorders such as cancer and photoallergic reactions. Natural products have been considered as non-irritate and potential sunscreen resources due to their UV absorption and anti-inflammatory activities. This study aimed to evaluate the in vitro ultraviolet radiation protective effect and anti-inflammatory activity of *K. galanga* rhizome oil and microemulsions. The chemical components of *K. galanga* rhizome oil was analyzed via gas chromatography coupled with mass spectrometry. Microemulsions containing *K. galanga* rhizome oil were formulated using a phase-titration method. The microemulsion was characterized for droplet size, polydispersity index, and zeta potential, using a dynamic light-scattering technique. The physical and chemical stability of the microemulsion were evaluated via a dynamic light scattering technique and UV-Vis spectrophotometry, respectively. The UV protection of *K. galanga* rhizome oil and its microemulsion were investigated using an ultraviolet transmittance analyzer. The protective effect of *K. galanga* rhizome oil against LPS-induced inflammation was investigated via MTT and nitric oxide inhibitory assays. In addition, a hydrogel containing *K. galanga* rhizome oil microemulsion was developed, stored for 90 days at 4, 30, and 45 °C, and characterized for viscosity, rheology, and pH. The chemical degradation of the main active compound in the microemulsion was analyzed via UV-Vis spectrophotometry. The formulated O/W microemulsion contained a high loading efficiency (101.24 ± 2.08%) of *K. galanga* rhizome oil, suggesting a successful delivery system of the oil. The size, polydispersity index, and zeta potential values of the microemulsion were optimized and found to be stable when stored at 4, 30, and 45 °C. *K. galanga* rhizome oil and microemulsion demonstrated moderate sun protective activity and reduced the nitric oxide production induced by LPS in macrophage cells, indicating that microemulsion containing *K. galanga* rhizome oil may help protect human skin from UV damage and inflammation. A hydrogel containing *K. galanga* rhizome oil microemulsion was developed as a topical preparation. The hydrogel showed good physical stability after heating and cooling cycles and long-term storage (3 months) at 4 °C. The use of *K. galanga* rhizome oil as a natural sun-protective substance may provide a protective effect against inflammation on the skin. *K. galanga* rhizome oil microemulsion was successfully incorporated into the hydrogel and has the potential to be used as a topical sunscreen preparation.

## 1. Introduction

Ultraviolet (UV) radiation is a form of electromagnetic radiation with a wavelength from 10 nm to 400 nm that is emitted by the sun and artificial sources such as halogen, fluorescent and incandescent lights, and lasers [1]. UV radiation is classified into three types, including ultraviolet A (UVA), ultraviolet B (UVB), and ultraviolet C (UVC), based on their wavelengths [2]. More than 95% of the UV radiation reaching the earth’s surface is UVA. UVA has longer wavelengths compared with UVB and UVC. Much of UVB can be absorbed by the ozone layer [3]. This type of UV radiation has a shorter wavelength. Hence, it can penetrate deeper layers of the epidermis. The skin is the body’s largest organ that acts as a protective barrier from mechanical, thermal, and physical injury and chemical agents. It prevents excessive loss of moisture and protein structure. Exposure of skin to UV radiation can frequently cause the skin to age, which is one of the main causes of skin damage. Long-term UV exposure causes skin wrinkles, dryness, roughness, reduced elasticity, inflammation, and skin cancer [4]. UV radiation, particularly UVB, can damage the epidermis’s DNA and protein structures, which is the outermost part of the skin. In addition, UVB triggers a local inflammatory state in the skin, subsequently stimulating counteracting immunosuppression. The long-term existence of an inflammatory microenvironment increases the risk of skin cancer and metastasis [5].

Several essential oils extracted from parts of the plants including almond oil, avocado, coconut, cottonseed, castor, olive, peanut, sesame, and soybean oils have been reported to absorb UV radiation [6]. The SPF value of olive oil and coconut oil was found to be approximately 8; castor oil, approximately 6; almond oil, approximately 5; mustard oil and chaulmoogra oil, approximately 3; and sesame oil, approximately 2 [6]. Sesame oil was claimed to block 30% of UV rays, whereas coconut, peanut, olive, and cottonseed oils resisted about 20% of UV light [7]. The use of photoprotective natural essential oils in sunscreen formulations would allow for a reduction in the number of chemical UV filters, reducing safety concerns, maintaining SPF, and meeting consumer demand for more natural products [7]. However, the concerns associated with natural sun protective action from natural oils are their instability and low bioavailability. Aromatic plant essential oils are rich in bioactive compounds such as terpenes, terpenoids, flavonoids, phenolics, and others. These constituents have varying physicochemical properties and stability, but they cannot be used in their pure form. As a result, a suitable delivery system is required to improve their bio-efficacy and stability during application.

*Kaempferia galanga* Linn. is a plant in the Zingiberaceae family cultivated in Southeast Asia countries. The pharmacological properties in the essential oil of *K.galanga* are attributed mostly to ethyl-cinnamate and ethyl-p-methoxy-cinnamate [8]. The essential oil extracted from the rhizome of *K. galanga* possesses moderate to high antifungal and anti-bacterial activities [9]. Das et al. reported antibacterial properties of *K. galanga* rhizome oil against both gram-positive and gram-negative [10]. Sun protective activity of *K.galanga* has been reported. However, the SPF values varied in the range of 4.51–4.69, which was low. In this study, the hydro distillation method with controlled extraction time and ratio of the plant and water was applied to obtain higher bioactive constituents.

Microemulsions are clear, optically isotropic, and thermodynamically stable systems generally composed of oil, water, and surfactant. Microemulsions are thermodynamically stable and form spontaneously or with very low energy input. Microemulsions require a high amount of surfactant compared to emulsions to maintain their thermodynamical stability [11]. Surfactants and co-surfactants stabilize the microemulsion system by decreasing the interfacial surface tension between two immiscible liquids, compensating for dispersion entropy and making the system thermodynamically stable [12]. A large amount of essential oil can be incorporated into the disperse phase of formulation [13,14]. Microemulsion can help solubilize lipophilic drugs and oil, enhances the absorption rate, and increases the bioavailability of the oil or encapsulated drugs. Microemulsion can protect the encapsulated drugs from hydrolysis and oxidation, depending on the type of surfactant. The presence of surfactant improves the cell membrane’s permeability, allowing for easier absorption. Addition of a surfactant decreases surface tension of the oil−water interface, thus stabilizing the connection between oil and the aqueous phases and enhancing the solubility of the oil. Thus, selection of the surfactant and cosurfactant is important for proper microemulsion formation [15]. Non-ionic surfactants such as polysorbate 80 are preferred to prepare microemulsions because they have high tolerability, low irritation and toxicity, and biocompatibility [16]. Polysorbate 80 or Tween 80 is a synthetic non-ionic surfactant consisting of fatty acid esters of polyoxyethylene sorbitan. Tween 80 contains both hydrophobic and hydrophilic moieties. The main function of Tween 80 as a surfactant is to lower the oil/water interfacial tension to very low values, allowing thermal motions to spontaneously disperse the two immiscible phases [17]. Tween 80 has been widely used as a surfactant to form microemulsion [13]. Pumival et al. have reported that Tween 80 alone can stabilize a microemulsion of *Citrus hystrix* leaf oil [13,14]. Setyawati et al. utilized Tween 80 as a surfactant to formulate a stable emulsion containing *K. galanga* rhizome oil [18]. 

Kundun et al. reported a nanoemulsion of *K. galanga* rhizome oil (0.4 mg/mL) prepared via an ultrasonication method using 1% saponin as a surfactant [19]. In another study, a nanoemulsion of *K. galanga* rhizome oil was prepared via ultrasonication using maltodextrin as a stabilizer and Tween 80 as a surfactant [18]. Praseptiangga et al. prepared an edible film incorporated with *Ceiba pentandra* honey and *K. galanga* oil [20]. *K. galanga* rhizome essential oil in a combination with *Alpinia malaccensis* rhizome oil was incorporated into the gel and demonstrated a significant topical carrageenan-induced anti-inflammatory activity in rats [21]. Athikomkulchai et al. reported an O/W cream containing 7% *w*/*w K. galanga* rhizome oil with a sun protection factor of 4.69 [22]. A cream containing a mixture of *K. galanga* rhizome extract and *Boesenbergia rotunda* at ratio of 8:2 and 7:3 had SPF values of 5.01 and 4.51, respectively [23]. *K. galanga* oil cream containing 1.2% of ethyl p-methoxycinnamate was shown to have antibacterial activity against *P. acne*, *S. aureus*, and *S. epidermidis* without inducing skin irritation in volunteers [24]. A sunscreen cream containing 10%, 20%, and 30% *Eucheuma cottonii* aqueous extract and *K. galanga* rhizome powder at a 1:1 ratio had SPF values of 8.8, 13.3, and 16.7, respectively. The authors suggested that *E. cottonii* and *K. galanga* have synergistic effects to be used as sunscreen agent [25].

A formulation, characterization, and stability study of a microemulsion of *K. galanga* rhizome oil has not been reported previously. Therefore, this study is the first report of developing a microemulsion system to carry *K. galanga* rhizome oil. In addition, the sun protective effect and anti-inflammatory activity of *K. galanga* rhizome oil incorporated in a nanosized-drug delivery system are demonstrated here for the first time. *K. galanga* rhizome oil microemulsion-based hydrogel was then prepared as a model gel preparation for topical use. 

The present study aimed to design a microemulsion system to deliver *K. galanga* rhizome oil and incorporate the microemulsion into a hydrogel preparation for topical use. The objective was to formulate a *K. galanga* rhizome oil microemulsion based on a pseudo-ternary phase diagram. The microemulsion was characterized by measuring the size, zeta potential, and polydispersity index (PDI). Physical and chemical stabilities of the microemulsion were tested. The in vitro photoprotective and anti-inflammatory activities of the microemulsion were evaluated. Hydrogel containing *K. galanga* rhizome oil microemulsion was formulated and characterized for its physical stability.

## 2. Results and Discussion

### 2.1. K. galanga Rhizome Oil Distillation Yield

The essential oil yield from the hydrodistillation of *K. galanga* rhizome oil was a clear pale yellow liquid with a strong and distinctive odor. The distillation of the *K. galanga* rhizome yielded 0.99 ± 0.09% *w*/*w* of essential oil. Sahoo et al. reported that the essential oil yield obtained from the hydrodistillation of *K. galanga* rhizome oil was 0.6% [26]. The in vitro cell cultured rhizomes yielded 0.9% essential oil. Bhuiyan et al. demonstrated that the yield of essential oils from the rhizomes of *K. galanga* from Bangladesh was 1.05% [27]. Srivastava et al. reported that *K. galanga* oil from rhizomes in India yielded 1.3% [28]. The higher % yield of essential oil might be due to the longer duration of hydrodistillation. In addition, the time of harvesting until extraction, plant storage, place of cultivation, and variation of genotypes might affect the yield of essential oil.

### 2.2. K. galanga Rhizome Oil Constituents Analyzed by GC-MS

GC-MS was used to analyze 35 compounds from *K. galanga* rhizome oil (Table 1). The most abundant volatile compounds in *K. galanga* rhizome oil were ethyl cinnamate (36.33%), (E)-ethyl-p-methoxycinnamate (23.77%), and pentadecane (13.12%). Wong et al. reported that the major constituents of the essential oil of *K. galanga* rhizomes growing in Malaysia were ethyl trans-p-methoxycinnamate (51.6%), ethyl cinnamate (16.5%), pentadecane (9.0%), 1,8-cineole (5.7%), γ-car-3-ene (3.3%), and borneol (2.7%) [29]. Bhuiyan reported that 2-ethyl (E)-4-ethoxy-3-methoxycinnamate (63.36%), ethyl cinnamate (6.31%), 4-cyclooctene-1-methanol (4.61%), caryophyllene oxide (4.37%), limonene (3.22%), borneol (2.46%), cubenol (1.67%), and nerolidyl acetate (1.05%) were predominantly found in oil from *K. galanga* rhizome cultivated in Bangladesh [27]. Trans-ethyl-*p*-methoxycinnamate (28.4–70.0%) and trans-ethyl cinnamate (11.5–26.6%) were the two most abundant compounds in rhizome oil in India. δ-3-carene (0.1–6.5%), 1,8-cineole (0.2–5.2%), borneol (1.0–2.4%), and pentadecane (6.0–16.5%) were also present in significant amounts [8]. Several factors may contribute to variations in the chemical composition of *K. galanga* rhizome essential oil, including the different geographic and environmental culture conditions such as temperature, humidity, and altitude of plant growth [30].

### 2.3. Quantitative Analysis of Ethyl Cinnamate in K. galanga Rhizome Oil via UV-Vis Spectrophotometry

The result from UV-Vis spectrophotometry showed that the highest absorbance value in the ethyl cinnamate spectrum was at a wavelength of 281 nm. The calibration curve of ethyl cinnamate was linear over the concentration range of 6 × 10^−5^ to 2 × 10^−3^% *v*/*v*. Each point on the curve was obtained from three replicates. The regression equation obtained was y = 1159.6x + 0.0343, with regression coefficient (R^2^) = 0.9994. The average concentration of ethyl cinnamate in *K. galanga* oil was 26.67 ± 0.59% of oil. 

### 2.4. Construction of the Pseudo-Ternary Phase Diagram

**Figure 1:**
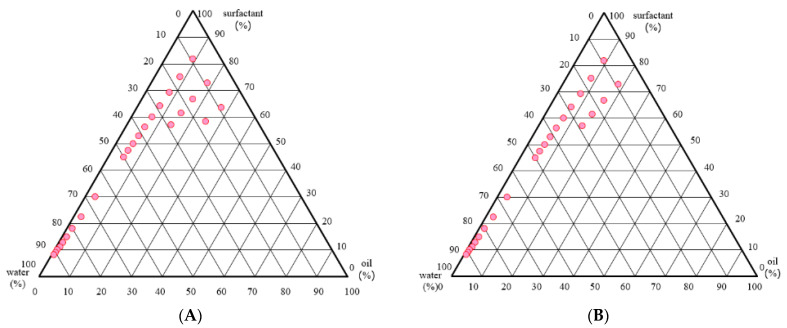

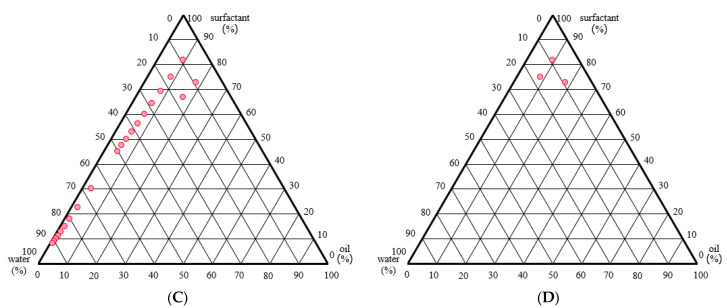
Effects of surfactant and co-surfactant on the microemulsion formation. The formulation contains (**A**) Tween 80^®^ only, (**B**) Tween^®^: propylene glycol at a ratio of 1:1, (**C**) Tween 80^®^: propylene glycol at a ratio of 1:2, and (**D**) Tween 80^®^: propylene glycol at a ratio of 1:3. The pink dots represented the region of microemulsion existence.

Pseudo-ternary phase diagrams were created by an increasing water content and decreasing Smix contents [13]. The effects of propylene glycol (PG) as a co-surfactant were studied by varying the ratios of Tween 80 and PG at 1:0, 1:1, 1:2, and 1:3. Figure 1 demonstrated the pseudo-ternary phase diagrams of *K. galanga* rhizome oil, S_mix_, and water-based microemulsion systems. According to the phase diagrams, the Tween 80/*K. galanga* rhizome oil/water system formed microemulsion regions with 9–25%, 9–18%, 9–17%, and 9–14% *K. galanga* rhizome oil at Tween 80:PG ratios of 1:0, 1:1, 1:2, and 1:3, respectively. The most optimal formulation contained 27.27% *K. galanga* rhizome oil, 63.64% Tween 80, and 9% water in microemulsion. These results indicated that increasing the Tween 80/PG ratio helps to increase the loading of *K. galanga* rhizome oil in the microemulsion. In this study, the *K. galanga* rhizome oil microemulsion could be formed with and without adding a co-surfactant. 

Pseudo-ternary phase diagrams were constructed to obtain the appropriate components and their concentration ranges that can result in a large existence area of microemulsion. An optimum proportion of an aqueous phase, an oil phase, and surfactant concentration can be adjusted based on the predictions made by the phase diagram. From the pseudoternary phase diagrams, it was concluded that the highest microemulsion zone was achieved for the microemulsions having a Tween 80:PG ratio of 1:0.

### 2.5. Type of K. galanga Rhizome Oil Microemulsion 

**Figure 2:**
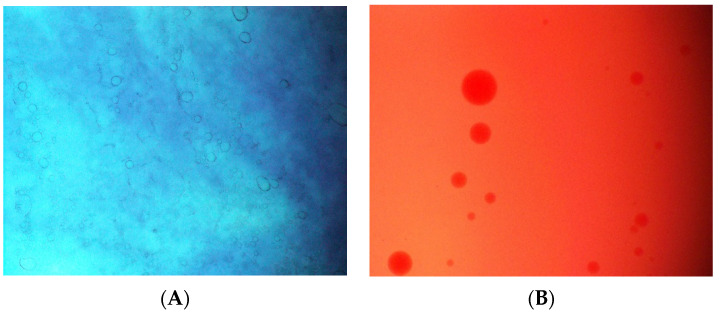

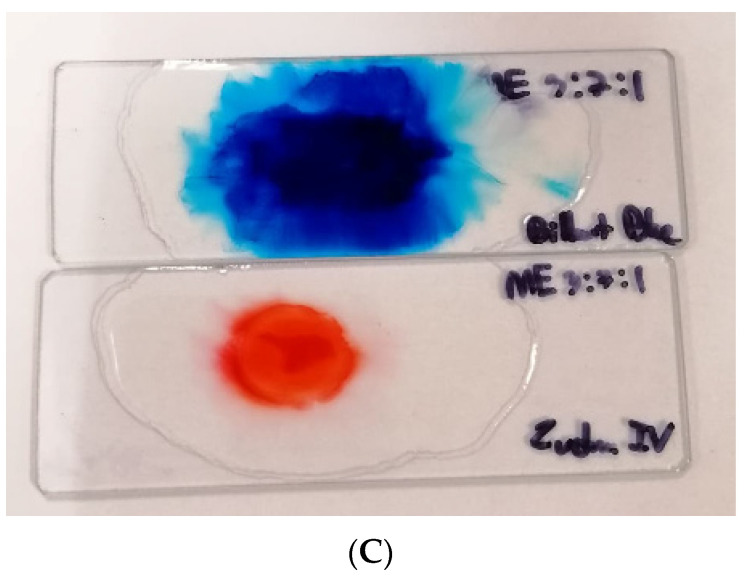
Identification of *K. galanga oil* microemulsion type. (**A**) Microemulsion stained with brilliant blue, (**B**) microemulsion stained with Sudan IV, and (**C**) diffusion of microemulsion in brilliant blue and Sudan IV. The micrographs were taken at a magnification power of 100×.

The microemulsion containing 27.27% *v*/*v* of *K. galanga* rhizome oil was selected for further characterization, type of microemulsion, a stability test, and a photoprotective test, as it contained the highest concentration of oil. The droplets of *K. galanga* rhizome oil microemulsion were stained with Sudan IV (Figure 2A), an oil soluble dye, whereas outside of the microemulsion, droplets were stained with brilliant blue (Figure 2B). In addition, the brilliant blue diffusion rate of *K. galanga* oil microemulsion was markedly increased compared with that of Sudan IV (Figure 2C). These results indicated that *K. galanga* rhizome oil was *o*/*w* microemulsion.

The electrical conductivity of *K. galanga* rhizome oil microemulsion was 5.44 ± 0.78 S/m. This result indicated that the *K. galanga* rhizome oil microemulsion developed in this study was o/w microemulsion since this type of microemulsion contained water in the continuous phase and resulted in significant conductivity. According to Singpanna et al., o/w microemulsion provided a relative higher conductivity compared with w/o microemulsion, with more than 10 µS/cm [31].

### 2.6. Physical Characteristics and Stability of Microemulsion

**Figure 3:**
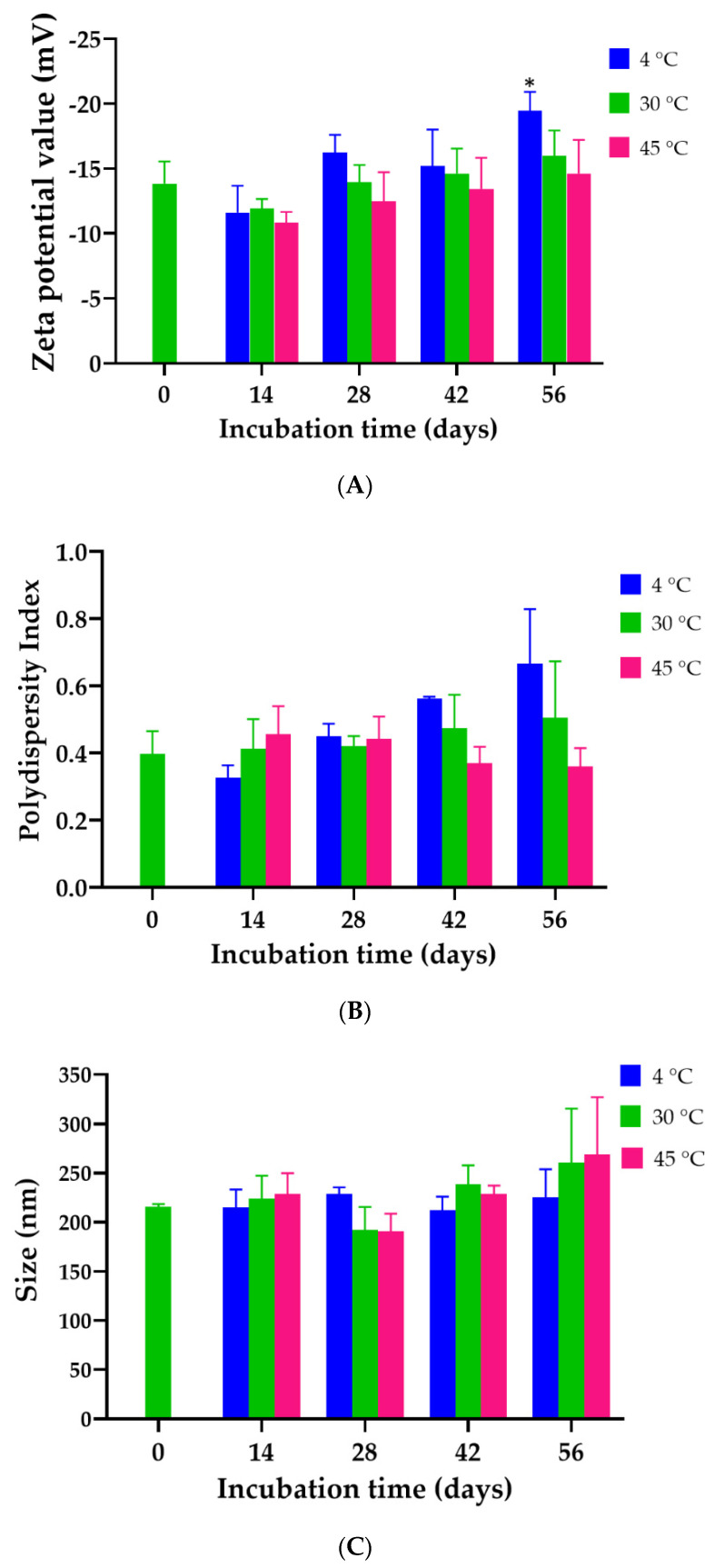
Physical stability of *K. galanga* rhizome oil microemulsion after storage at 4, 30, and 45 °C for 56 days. (**A**) Droplet size, (**B**) polydispersity index, and (**C**) zeta potential values. The results represent the mean ± SD of three experiments. * indicates *p* < 0.05 compared with Day 0.

The droplet size of the freshly prepared *K. galanga* rhizome oil microemulsion was 215.9 ± 2.50 nm. The PDI and zeta potential of the microemulsion were 0.397 ± 0.068 and −13.8 ± 1.7 mV, respectively. The physical stability of the *K. galanga* rhizome oil microemulsion was evaluated by observing changes in particle size, size distribution, and zeta potential values over 56 days of storage at 4 °C, 30 °C, and 45 °C. The evaluations were carried out to uncover the physical stability of the formulation containing 27.27% *K. galanga* rhizome oil, 63.64% Tween 80, and 9% water. The results indicated that the size and PDI of the *K. galanga* rhizome oil microemulsion were not influenced by time and storage temperature, as shown in Figure 3. The size of the microemulsion on day 28 seemed to be smaller, but the average size was not significantly different from the baseline (Day 0). The reason for the size reduction might be due to the diffusion of oil from the microemulsion [32]. The results of the average size, PDI, and zeta potential values of the microemulsion on days 0, 14, 28, and 56 displayed no statistically significant differences at storage temperatures of 4 °C, 30 °C, and 45 °C. The zeta potential is one of the key properties of the particle that can affect the particle stability as well as its cell adhesion. Zeta potential (positive or negative) values have a significant role in stabilizing particle suspension. This is attributed to the electrostatic repulsion between particles with the same electric charge that causes segregation of the particles [33]. These results showed that the developed *K. galanga* rhizome oil microemulsion was highly stable when stored at 4, 30, and 45 °C. The high stability of the microemulsion suggests a thermodynamically stable system. The pH of the microemulsion was 6.73 ± 0.04, indicating that it had a neutral pH.

### 2.7. Chemical Characterization of K. galanga Rhizome Oil and Microemulsion and Chemical Stability of K. galanga Rhizome Oil Microemulsion 

**Figure 4:**
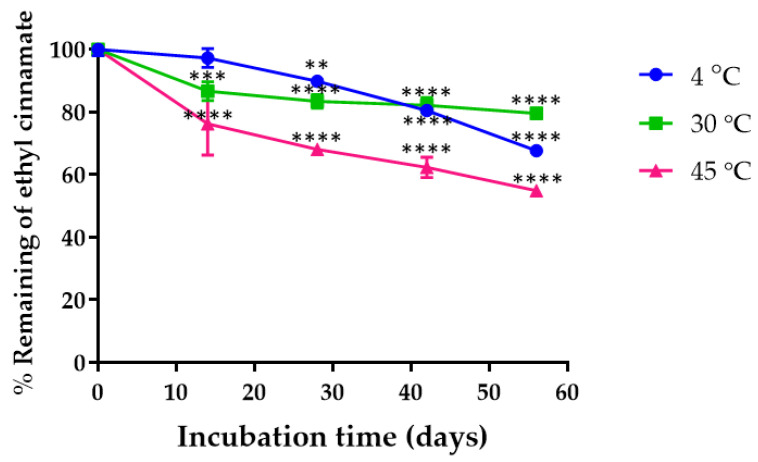
%Remaining of ethyl cinnamate after storage at 4, 30, and 45 °C for 0, 14, 28, 42, and 56 days. **, ***, and **** indicate *p* < 0.01, *p* < 0.001, and *p* < 0.0001.

The UV-Vis spectrophotometry results revealed that *K. galanga* rhizome oil contained 26.67 ± 0.58% of ethyl cinnamate in the oil. Since 27.27% of oil was included in the microemulsion formulation, theoretically, 7.27% of ethyl cinnamate was present in the microemulsion. The results showed that the % recovery of ethyl cinnamate in the microemulsion was 101.24 ± 2.08%, suggesting a successful loading of *K. galanga* rhizome oil in the microemulsion system. The chemical stability of ethyl cinnamate in *K. galanga* rhizome oil microemulsion is shown in Figure 4. The remaining percentage of ethyl cinnamate in the microemulsion stored at 4, 30, and 45 °C gradually decreased over 56 days. After storage for 56 days, the amount of ethyl cinnamate stored at 4 °C, 30 °C, and 45 °C was 67.61 ± 0.75%, 79.58 ± 0.52%, and 54.85 ± 0.76%, respectively. Many types of oils could be degraded at low temperature due to crystallization of the oil phase. The crystallization process of the oil phase in microemulsion led to an increase in the energy at the oil and water interface and destabilized the microemulsion. This process might lead to the possibility of destabilization of the oil and active compound existing in the surrounding aqueous phase [34]. The reduction in ethyl cinnamate in *K. galanga* rhizome oil microemulsions may be due to chemical degradation. The major degradation pathway of ethyl cinnamate is hydrolysis reaction. Singh reported that the specific rate constant of hydrolysis decreased when the percentage of co-solvent increased. This was due to the decrease in the dielectric constant or less polarity of the reaction medium, resulting in the stabilizing of the ethyl cinnamate and hence increasing the Gibb’s free energy. Therefore, incorporating *K. galanga* rhizome oil containing ethyl cinnamate may enhance the chemical stability of the active compound due to the low amount of water in the formulation [35]. Tween 80 has a dielectric constant value of 8.75, while the dielectric constant value of water is 78 [36]. The dielectric constant of the Tween 80 and water mixture in the microemulsion system was 17.46, which is lower than that of the aqueous system. Therefore, this system may reduce the hydrolysis rate of ethyl cinnamate and increase the chemical stability of the active compound.

### 2.8. In Vitro Photoprotective Effect of K. galanga Rhizome Oil and Microemulsion

The UVB protection efficacy assessment of *K. galanga* rhizome oil and microemulsion containing 27.27% of the oil revealed that the sun protection factor (SPF) of *K. galanga* rhizome oil and microemulsion was 33.34 ± 1.18 and 18.60 ± 2.71, respectively, suggesting high and moderate sun protection as classified by the US FDA. The microemulsion base without *K. galanga* rhizome oil showed an SPF value of 0.92 ± 0.00, indicating that the sun protection effect of *K. galanga* rhizome microemulsion was attributed to the oil. The critical wavelengths of *K. galanga* rhizome oil and microemulsion were 337.78 ± 0.67 and 337.00 ± 0.00 nm, respectively. The critical wavelength (λc) is the wavelength at which the absorbance spectrum reached 90% of the total integral from a wavelength at 290–400 nm. Since the US FDA specified that a broad-spectrum sunscreen must offer a critical wavelength greater than 370 nm, *K. galanga* rhizome oil and microemulsion were not considered a broad-spectrum sunscreen.

### 2.9. In Vitro Cytotoxicity of K. galanga Rhizome Oil and Microemulsion on RAW 264.7 Cells

**Figure 5:**
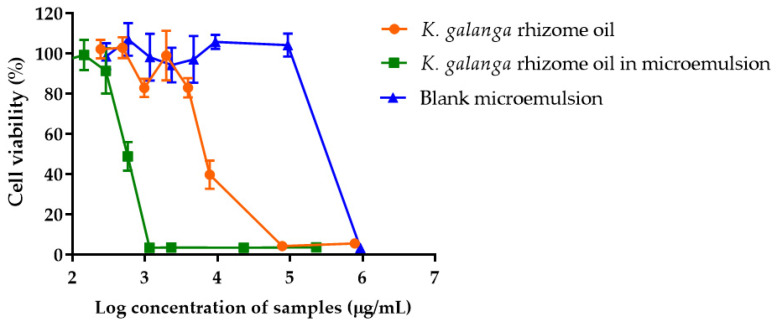
Cell viability of RAW 264.7 after the treatment with *K. galanga* rhizome oil, *K. galanga* rhizome oil microemulsion, and blank microemulsion for 24 h.

To examine the effect of *K. galanga* rhizome oil and microemulsion on macrophage cells, RAW 264.7 cell lines were treated with the oil and microemulsion for 24 h and cell viability was determined using MTT assay. The cells exposed to blank microemulsion at a concentration up to 9.3 mg/mL showed a % cell viability of 98.62 ± 0.72% suggesting the safety of blank microemulsion. The cytotoxicity of *K. galanga* rhizome oil and microemulsion increased in a dose-dependent manner (Figure 5). *K. galanga* oil and microemulsion containing *K. galanga* oil at concentrations of 0.24–3.92 mg/mL and 0.07–0.29 mg/mL, respectively, showed no cytotoxicity to RAW 264.7 cells. The IC_50_ values of *K. galanga* rhizome oil and microemulsion were 6.8 mg/mL and 0.6 mg/mL, respectively. A previous study reported IC_50_ values of 100.60 μg/mL, 83.04 μg/mL, and 94.75 μg/mL for ethyl-cinnamate, ethyl-p-methoxy-cinnamate, and trans-cinnamaldehyde, respectively [37] These results suggested that the microemulsion enhanced the cytotoxicity of *K. galanga* rhizome oil to RAW 264.7 cells, which was probably due to the enhancing solubility and penetration of the oil through the cell membrane via the surfactant of the microemulsion [38,39,40]. The cytotoxicity criteria of *K. galanga* rhizome oil, microemulsion containing oil, and blank microemulsion against RAW 264.7 cells conformed with ISO 10993–5:2009, which describes that reduction of cell viability by more than 30% is considered a cytotoxic effect.

### 2.10. Anti-Inflammatory Effects of K. galanga Rhizome Oil and Microemulsion

**Figure 6:**
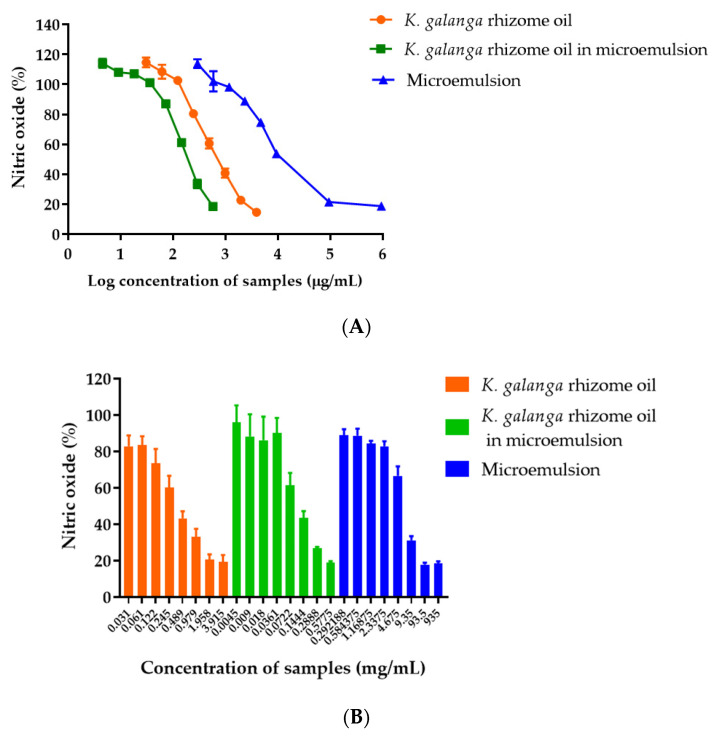

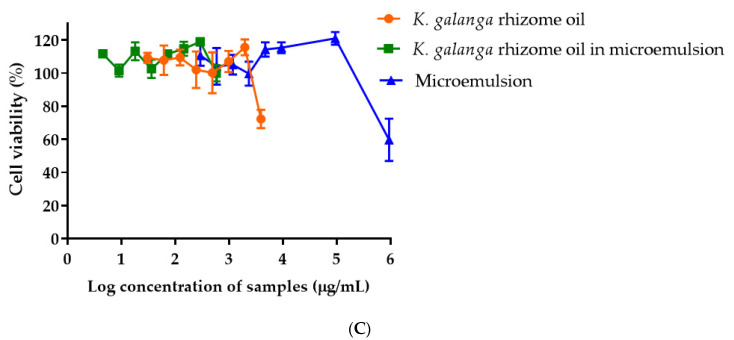
(**A**) Nitric oxide secretion (%) of RAW 264.7 after the treatment with *K. galanga* rhizome oil, *K. galanga* rhizome oil microemulsion, and blank microemulsion for 18 h, compared with LPS-induced RAW 264.7 cells, (**B**) plots of nitric oxide secretion (%) of RAW 264.7 vs. concentrations of treated samples, and (**C**) cell viability of RAW 264.7 after the treatment with *K. galanga* rhizome oil, *K. galanga* rhizome oil microemulsion, and blank microemulsion after nitric oxide induction with LPS.

Natural anti-inflammatory agents have gained significant interest in the treatment of inflammation due to several limitations and side effects of steroids and NSAIDS. *K. galanga* rhizome oil and microemulsion at non-cytotoxic doses were tested for anti-inflammatory activity using NO inhibition against LPS-stimulated RAW 264.7 cells using the Griess assay. The results showed that RAW 264.7 cells induced with LPS produced large amounts of NO (100%) compared with untreated cells, whereas cells treated with *K. galanga* rhizome oil and microemulsion significantly decreased NO levels. *K. galanga* rhizome oil and microemulsion revealed a dose-dependent nitric oxide reduction (Figure 6A). The IC_50_ values of *K. galanga* rhizome oil, *K. galanga* rhizome oil microemulsion, and blank microemulsion that can reduce 50% of nitric oxide secretion were 770.5, 203, and 16,672 µg/mL, respectively. The results demonstrated that microemulsion enhanced the nitric oxide reduction effect of *K. galanga* rhizome oil. This might be due to the presence of surfactant within the microemulsion, which may enhance penetration of the oil into the RAW 264.7 cells. Several mechanisms have been proposed to explain the penetration-enhancing effect of microemulsions. The first reason is the small droplet size and large surface area/volume ratio of the microemulsion. The second reason is attributed to the surfactants that can diffuse to the cell surface and increase the permeation of oil, either by disrupting the lipid structure of the cell membrane or by increasing the partition coefficient of the oil between the cell membrane and the medium. The structural organization of the oil and aqueous phases and the presence of the surfactant-containing interface create additional solubility regions, increasing the loading capacity of microemulsions and enhanced oil penetration through the cell membrane [41]. 

Ethyl-cinnamate and ethyl-p-methoxy-cinnamate are considered potent anti-inflammatory constituents of *K. galanga* oil that significantly inhibit pro-inflammatory cytokines such as COX-1, COX-2, IL-1, and TNF-α [42]. A previous study reported that hydro-alcoholic root extract of *Alpinia galanga* downregulates the release of pro-inflammatory mediators and vital enzymes of inflammation that stimulate the release of anti-inflammatory intermediaries in LPS-stimulated RAW 264.7 cells [43]. To validate that the decrease in NO production was not due to the death of the RAW 264.6 cells exposed to the *K. galanga* rhizome oil, *K. galanga* oil microemulsion, and blank microemulsion, a cell viability test was carried out. The cytotoxicity assay showed up to 1.96 mg/mL of *K. galanga* rhizome oil, 0.58 mg/mL of *K. galanga* rhizome in microemulsion, and 93.5 mg/mL of blank microemulsion, and the % cell viability was more than 80% (Figure 6B). The results indicated that within this range, none of the samples showed any cytotoxic effects towards RAW 264.7 cells [44,45]. The results suggested that the low nitric oxide production in the treated cell was not caused by cell death but was due to nitric oxide inhibition by the tested samples.

The increase in viability of RAW 264.7 macrophage cells to be higher than 100% might be due to the proliferation-enhancing effect of *K. Galanga* rhizome oil. Sugiartanti et al. showed that the ethanolic extract of *K. galanga* rhizome significantly stimulated the proliferation of splenocytes, indicating immunomodulatory activity of the extract [46]. Dash et al. also reported that *K. galanga* rhizome oil also showed an increase in lymphocyte proliferation in a dose-dependent manner, suggesting the immunomodulatory potentialities of the essential oil [47]. The bioactive compounds responsible for the proliferation of lymphocytes and splenocytes might be p-methoxycinnamate in the essential oil. The mechanism of *K. galanga* rhizome oil to enhance immune cell proliferation might be due to a mitogenic-inducing effect [48]. 

### 2.11. Formulation of K. galanga Rhizome Oil Microemulsion Based Hydrogel

**Figure 7:**
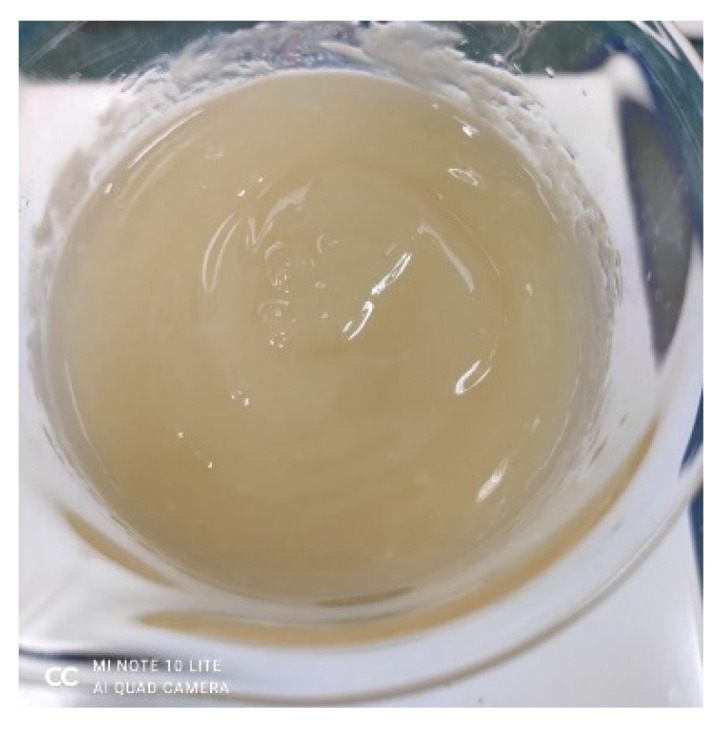
Appearance of *K. galanga* rhizome oil microemulsion-based hydrogel.

As *K. galanga* rhizome oil microemulsion had an SPF value of 18.60 ± 2.71, the concentration of the oil in the hydrogel formulation was suggested to be at least 80.65% *w*/*w* to ensure that the SPF of the hydrogel was at a moderate level (SPF = 15–30). The hydrogel containing 85% *w*/*w* of *K. galanga* rhizome oil microemulsion was formulated using sodium carboxymethyl cellulose as a gelling agent with different concentrations. The results showed that the viscosity of the product was increased when the concentration of SCMC increased. Hydrogel containing *K. galanga* rhizome oil microemulsion prepared using 1% *w*/*w* SCMC as a gelling agent was the best formulation and was then further characterized (Figure 7).

Gels and hydrogels are interchangeable terms for polymeric cross-linked network structures. Gels are defined as a significantly dilute cross-linked system that might be weak or strong based on their steady-state flow behavior. The term hydrogel refers to three-dimensional network structures made of a synthetic and/or natural polymer capable of absorbing and retaining large amounts of water. When a polymeric network is hydrated in an aqueous environment, the network’s hydrophilic groups or domains form a hydrogel structure. Hydrogels are broadly classified into two categories, which are permanent (chemical) gel and reversible (physical) gel. The hydrogels using SCMC as a gelling agent in this study were formed when the networks were held together by molecular entanglements and with secondary forces including ionic or hydrogen bonding or hydrophobic interactions.

### 2.12. Physical Stability of K. galanga Rhizome Oil Microemulsion Based Hydrogel

**Figure 8:**
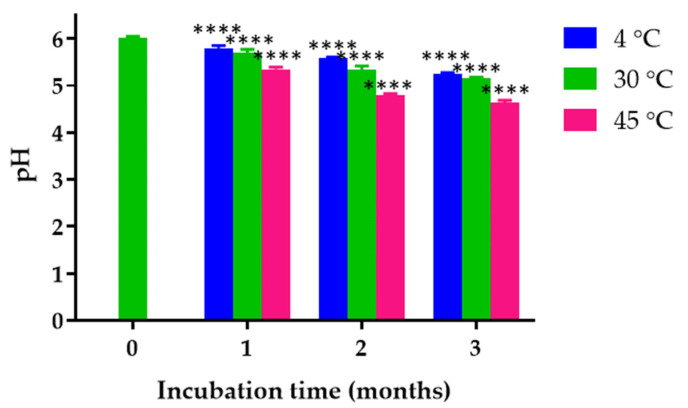
Effects of long-term storage on the pH of *K. galanga* rhizome oil microemulsion-based hydrogel after storage at 4, 30, and 45 °C for 3 months. **** indicates *p* < 0.001.

The pH of the freshly prepared *K. galanga* rhizome oil microemulsion-based hydrogel was 6.01 ± 0.04. The pH of the hydrogel stored at 4 °C for 30, 60, and 90 days significantly decreased, as shown in Figure 8. Regarding the heating/cooling cycle study, the pH value after heating/cooling cycle tests reduced to 5.74 ± 0.04. However, the pH of the microemulsion was in an acceptable range. Lukic et al. reported that topical products should be acidified and possess pH values in the range of 4 to 6 [49]. The results suggested that incubation time and temperature affected the pH values of the *K. galanga* rhizome oil microemulsion-based hydrogel. The decrease in pH values might have resulted from the degradation product of ethyl cinnamate and other compounds in *K. galanga* rhizome oil. Ethyl cinnamate is an ester of cinnamic acid and ethyl alcohol. It can be hydrolyzed to cinnamic acid, which lowers the pH value of the medium [50]. Ethyl cinnamate was degraded by alkaline-catalyzed hydrolysis. Our results were confirmed by the previous reports showing that the specific rate constant of ethyl cinnamate hydrolysis reaction depends on the storage temperature [51,52,53]. 

**Figure 9:**
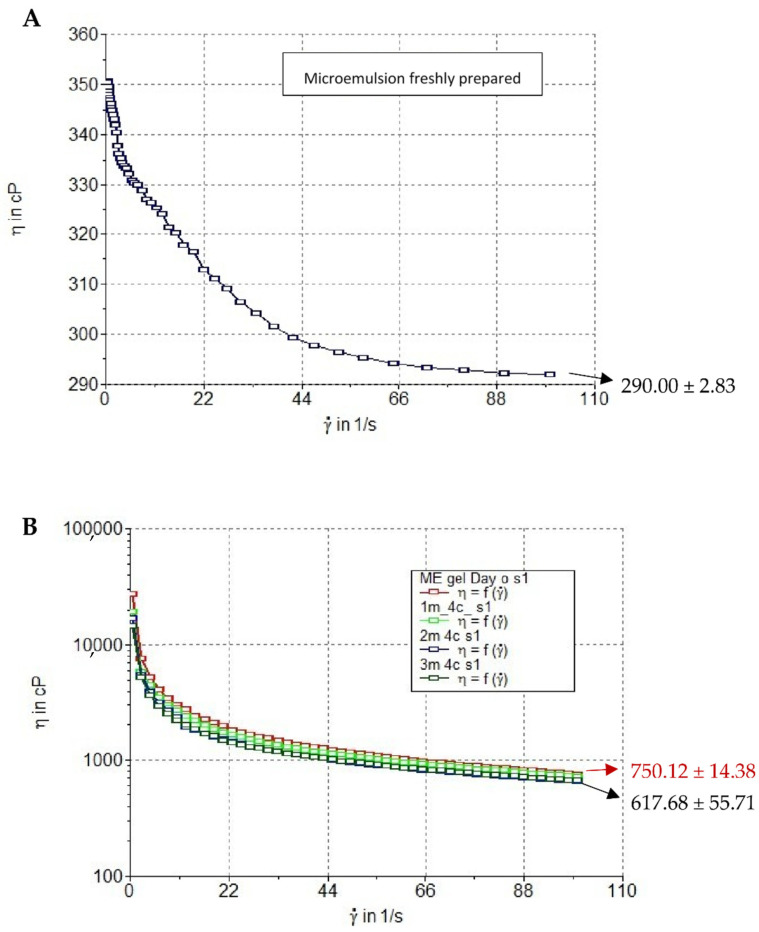

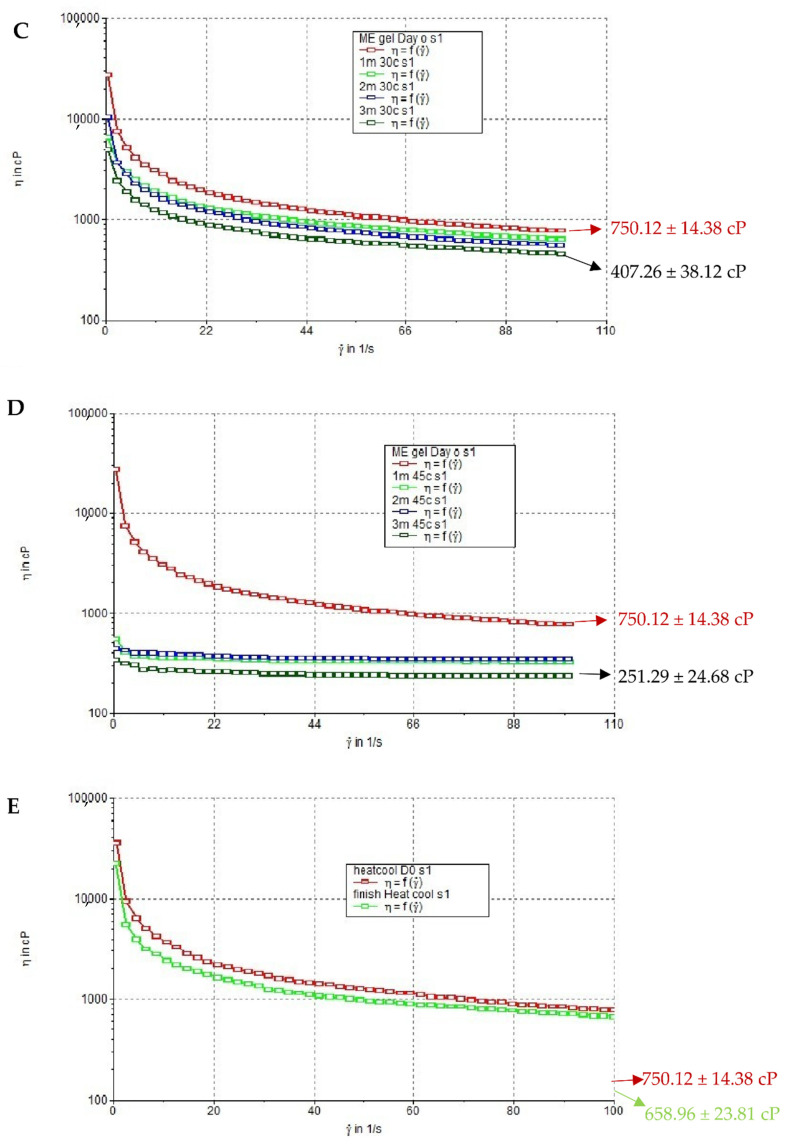

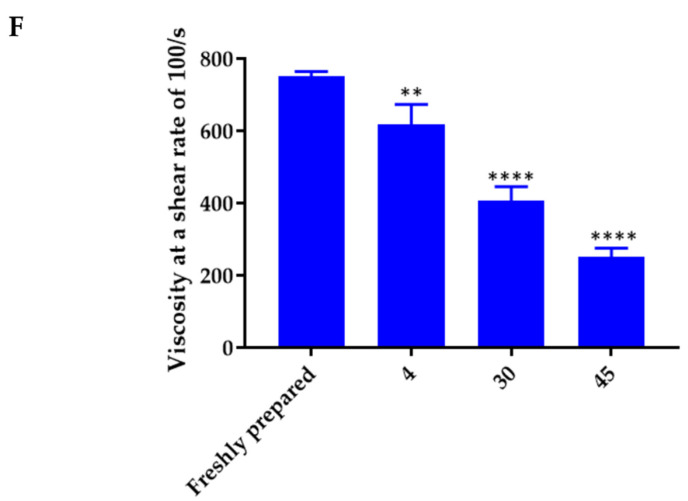
Rheology behavior of (**A**) *K. galanga* rhizome oil microemulsion and *K. galanga* rhizome oil microemulsion-based hydrogel after storage at (**B**) 4 °C, (**C**) 30 °C, (**D**) 45 °C, and (**E**) 6 heating/cooling cycles and (**F**) viscosity of hydrogel at shear rate of 100/s after 3-month storage. ** and **** indicate *p* < 0.01 and *p* < 0.0001.

The viscosity and rheology of *K. galanga* rhizome oil microemulsion and hydrogel was measured using a rheometer. The rheology diagram of the microemulsion and the hydrogel in Figure 9A–D indicates that the *K. galanga* rhizome oil microemulsion and hydrogel had pseudoplastic flow properties. The viscosity of the *K. galanga* rhizome oil microemulsion at a shear rate of 0.5, 20, 40, 60, 80, and 100 s^−1^ were 346.00 ± 1.41, 314.50 ± 2.12, 296.50 ± 3.54, 292.50 ± 3.54, 290.5 ± 2.12, and 290.00 ± 2.83. The viscosity values of the hydrogel measured at 25 °C after fresh preparation at a shear rate of 0.5, 20, 40, 60, 80, and 100 s^−1^ were 23,678.84 ± 5114.17, 2172.03 ± 129.29, 1358.28 ± 42.05, 1053.95 ± 26.28, 869.19 ± 15.28, and 750.12 ± 14.38 cP, respectively. The viscosity of the hydrogel depended on the storage time and temperature. The apparent viscosity of SCMC hydrogel is a function of temperature and shear rate. The SCMC polymer is formed via electrostatic interactions of hydrogen bonding. High temperature can break these bonds to an equilibrium state. The equilibrium corresponds to an activation energy value, determining the temperature dependence of the viscosity [54]. The viscosity of hydrogels stored at 4, 30, and 45 °C for 90 days was significantly reduced after applying a shear rate of 100 s^−1^ (Figure 9D). The viscosity of microemulsion-based hydrogel after six heating/cooling cycles was reduced to 658.96 ± 23.81 cP at a shear rate of 100 s^−1^. These results suggest that *K. galanga* rhizome oil microemulsion-based hydrogel should be stored at 4 °C.

## 3. Conclusions

In this study, the chemical composition of *K. galanga* rhizome oil was determined using GC-MS. The results showed that ethyl cinnamate was the major constituent of the essential oil. Microemulsion of the oil was prepared using Tween 80 as a surfactant. *K. galanga* rhizome oil and microemulsion exhibited moderate in vitro sun protective and anti-inflammatory activities against macrophage cells. *K. galanga* rhizome oil microemulsion-based hydrogel was successfully prepared using sodium carboxymethyl cellulose as a gelling agent. The hydrogel containing microemulsion had pseudoplastic flow and an optimal pH value suitable for skin application. The stability study of the microemulsion and hydrogel revealed that the hydrogel containing *K. galanga* rhizome oil should be stored at 4 °C.

## 4. Materials and Methods

### 4.1. Materials

Ethyl cinnamate analytical standard, sulfanilamide, N-(1-naphthyl) ethylenediamine, lipopolysaccharide, and sodium nitrite were purchased from Sigma-Aldrich (St. Louis, MO, USA). Polyoxyethylene 80 (Tween 80) and propylene glycol (PG) were obtained from NSG Nam Siang Co. Ltd. (Bangkok, Thailand). Dimethyl sulfoxide was supplied Thermo Fisher Scientific (Waltham, MA, USA). A RAW 246.7 cell line was provided by the American Type Culture Collection (Manassas, VA, USA). Dulbecco’s modified Eagle’s medium (DMEM), fetal bovine serum, and penicillin-streptomycin were obtained from Thermo Fisher Scientific (Carlsbad, CA, USA). 

### 4.2. Plant Collection and Identification

Rhizomes of *K. galanga* were purchased from V.P. Pharmacy Co., Ltd. (Bangkok, Thailand). The specimen was identified by Assistant Professor Sirivan Athikomkulchai and kept at the Faculty of Pharmacy, Srinakharinwirot University, Nakhonnayok, Thailand.

### 4.3. Hydrodistillation of K. galanga Rhizome

Dried *K. galanga* rhizomes were washed, allowed to dry for 6 h at ambient temperature, and then pulverized using mortar and pestle. Dried *K. Galanga* rhizome (300 g) underwent hydrodistillation with a Clevenger-type apparatus [55]. The rhizome of *K. galanga* was immersed in 3.5 L of distilled water. The extraction was carried out in a 5 L flask for 3 h until the amount of essential oil was stabilized. The moisture in essential oil was absorbed using sodium sulfate. The oil was stored in amber glass bottles at 2–8 °C until use. The yield of the essential oil was calculated using Equation (1).

(1) $$Yield\;\left(\%\right)=\frac{Weight\;of\;oil\;obtained\;from\;distillation\;\left(g\right)}{Weight\;of\;dried\;K.\;Galanga\;\mathrm{rhizome}\;\left(g\right)}\times 100\%$$

### 4.4. Chemical Analysis of K. galanga Rhizome Oil Constituents via Gas Chromatography Coupled with Mass Spectrometry

*K. galanga* rhizome oil composition was determined via gas chromatography-mass spectrometry (GC-MS) analysis (Agilent, gas spectrometer model 7890 B and mass spectrometer model 5977 B, Agilent Technologies, Inc., Santa Clara, CA, USA). The *K. galanga* rhizome oil sample was prepared at a concentration of 9.6 mg/mL in hexane. The oil was injected into a column HP-5M5 UI (30 m. × 0.25 mm. i.d.; 0.25 µm film thickness) with an injection volume of 0.5 µL. The GC-MS spectrum was obtained using helium as a carrier gas with a flow rate of 1.0 mL/min. The column temperature progressed from 60 °C, held for 1 min, then increased to 180 °C (at a rate of 10 °C/min), and increased to 280 °C (at a rate of 20 °C/min). The total run time was 39 min. The selected mass range was scanned between 30 to 550 amu. The chemical constituents were identified by comparing their MS to those in the NISTO5 library and those reported in the literature. The mass spectra library and percentage composition were calculated using GC peak areas.

### 4.5. Chemical Characterization of K. galanga Rhizome Oil by UV-Vis Spectrophotometry

A standard solution of ethyl cinnamate with concentrations in the range of 6 × 10^−5^–1.0% *v*/*v* in DMSO was prepared via two-fold dilution. The absorbance of ethyl cinnamate-containing solutions was measured using UV-Vis spectrophotometry in the UV range of 200–400 nm with DMSO as a blank [56]. The UV spectra of ethyl cinnamate at various concentrations were obtained to establish the wavelength of the maximum absorbance. The concentration of ethyl cinnamate in *K. galanga* rhizome oil was determined via UV-Vis spectrophotometry. *K. galanga* rhizome oil (10 µL) was dissolved in DMSO (990 µL) and diluted using DMSO to double fold to obtain oil concentrations in the range of 6 × 10^−5^–1.0% *v*/*v*. The absorbance of *K. galanga* rhizome oil was read at the wavelength of maximum absorbance for ethyl cinnamate. The concentration of ethyl cinnamate was calculated based on the calibration curve of ethyl cinnamate. Three concentrations of *K. galanga* oil were prepared in DMSO and the absorbance measured at a maximum absorption wavelength using UV-Vis spectrophotometry (Shimadzu Corp., Kyoto, Japan). The concentration of ethyl cinnamate in the oil was calculated based on the ethyl cinnamate standard curve.

### 4.6. Preparation of K. galanga Rhizome Oil Microemulsion by the Phase Titration Method

Microemulsion was formulated using pseudo-ternary phase diagrams of *K. galanga* rhizome oil, surfactant, co-surfactant, and water. The optimal ratio and concentration of each component that formed a clear microemulsion were obtained from a water titration method [13]. Polysorbate 80 (Tween 80^®^) and propylene glycol were mixed in fixed-weight ratios of 1:0, 1:1, 1:2, and 1:3. The surfactant mixtures (S_mix_) were added to *K. galanga* rhizome oil with various ratios of oil to S_mix_: 1:9, 2:8, 3:7, 4:6, 5:5, 6:4, 7:3, 8:2, and 9:1 (*v*/*v*). Each composition was added with ultrapure water 100 µL/time (10 times), followed by adding 1000 µL of water until the total volume reached 10,000 mL. The microemulsions containing *K. galanga* rhizome oil were obtained by vigorously stirring the mixtures at ambient temperature. The formation of a microemulsion was revealed by visual examination for transparency. The S_mix_, oil, and water ratios that resulted in transparent mixtures were represented as pink points on the phase diagram. The microemulsion’s transparency was checked again after 24 h.

### 4.7. Measurement of Size, PDI, and Zeta Potential Values of K. galanga Rhizome Oil Microemulsion

The hydrodynamic diameter, PDI, and zeta potential values of *K. galanga* rhizome oil microemulsion were measured using a Zetasizer Nano ZS with a dynamic light scattering technique (Malvern Panalytical, Bristol, UK). At a 173° scattering angle and 25 °C, the effective hydrodynamic diameter and PDI were measured. [14].

### 4.8. Type of K. galanga Rhizome Oil Microemulsion 

The type of microemulsion was identified by the staining method. The water-soluble dye (Brilliant blue) and oil soluble dye (Sudan IV) were used to evaluate the microemulsions’ type by staining the microemulsion droplets with the dye. *K. galanga* rhizome oil microemulsion stained with the dye was observed under a light microscope at 100× magnification (Olympus BX53, Olympus-Life Sciences, Tokyo, Japan). The micrographs of the microemulsion were imaged using Cellsens standard software (Olympus-Life Sciences, Tokyo, Japan). The type of microemulsion was confirmed by observing the miscibility of the microemulsion and dye. *K. galanga* rhizome oil microemulsion (1 mL) was placed on the glass slide. Brilliant blue or Sudan IV solutions (1 mL) were then dropped on the microemulsion. The diffusion rates of *K. galanga* rhizome oil microemulsion were visualized for 1 min. Sudan IV diffusion rate would be greater than that of brilliant blue when the microemulsion was of the water in oil type. When the microemulsion was oil in water, the diffusion rate of brilliant blue was higher than that of Sudan IV.

The type of *K. galanga* rhizome oil microemulsion was confirmed via electrical conductivity measurement. Electrical conductivity measurement can help differentiate w/o microemulsion, o/w microemulsion, bicontinuous, and solution-type microemulsions [57]. Electrical conductivity of K. galanga rhizome oil microemulsion was measured using digital multimeter (Sanwa Electric Industry Co., Ltd., Tokyo, Japan). Measurements were carried out in triplicate at 25 °C.

### 4.9. Determination of the Physical Stability of K. galanga Rhizome Oil Microemulsion

*K.* galanga rhizome oil microemulsion was stored in light-protected glass vials for 56 days at 4 °C, 30 °C, and 45 °C. The size, PDI, and zeta-potential values of samples were determined using a dynamic light scattering technique at various storage times (0, 14, 28, 42, and 56 days) (Malvern Panalytical, Bristol, United Kingdom).

### 4.10. Determination of % Recovery of Ethyl Cinnamate in K. galanga Rhizome Oil Microemulsion

UV-Vis spectrophotometry was used to determine the percentage recovery of ethyl cinnamate loaded in *K. galanga* rhizome oil microemulsion. The microemulsion of *K. galanga* rhizome oil was dissolved in DMSO by adding 10 µL of microemulsion to 990 µL of DMSO. In the absence of the *K. galanga* rhizome oil microemulsion, the components used to formulate the microemulsion were diluted in DMSO and used as a blank for UV-Vis spectrophotometry. The amount of ethyl cinnamate in *K. galanga* rhizome oil microemulsion was measured using a double beam UV-1700 UV-Vis spectrophotometer at a maximum wavelength of 281 nm (Shimadzu Corp. Kyoto, Japan). Using standard calibration data, the amount of ethyl cinnamate in the microemulsion was calculated. The recovery percentage of ethyl cinnamate in the microemulsion was calculated using Equation (2).

(2) $$\%\;Recovery\;of\;ethyl\;cinnamate\;in\;microemulsion=\frac{Amount\;of\;ethyl\;cinnamate\;found\;in\;microemulsion}{Amount\;of\;ethyl\;cinnamate\;added\;in\;microemulsion}\times 100\%$$

### 4.11. Chemical Stability of Ethyl Cinnamate in K. galanga Rhizome Oil Microemulsion

The chemical stability of ethyl cinnamate in *K. galanga* rhizome oil microemulsion was evaluated by measuring the change in ethyl cinnamate concentration during storage. *K. galanga* rhizome oil microemulsion was stored in light-protected glass vials for 56 days at 4 °C, 30 °C, and 45 °C. The samples were collected at predetermined time intervals (0, 14, 42, and 56 days). To dissolve the ethyl cinnamate, the microemulsions were diluted 100 times in DMSO (10 µL of microemulsion diluted in 990 µL of DMSO). As a control, a microemulsion without *K. galanga* rhizome oil was used. By dissolving the ethyl cinnamate standard in DMSO in a range of 0.00006–1% *v*/*v*, a calibration curve was created. The remaining ethyl cinnamate in *K. galanga* rhizome oil microemulsion was determined by measuring absorbance at the maximum wavelength of 281 nm with a double beam UV-1700 UV-Vis spectrophotometer (Shimadzu Corp., Kyoto, Japan). Using standard calibration data, the amount of ethyl cinnamate in the microemulsion was calculated. Equation (3) was used to calculate the percentage of remaining ethyl cinnamate in the microemulsion.

(3) $$\%\;Remaining\;of\;ethyl\;cinnamate\;in\;microemulsion\;\;\;\;\;\;\;\;\;\;\;\;\;\;\;\;\;\;\;\;\;\;=\frac{Amount\;of\;ethyl\;cinnamate\;remaining\;in\;microemulsion}{Amount\;of\;ethyl\;cinnamate\;in\;freshly\;prepared\;microemulsion}\times 100$$

### 4.12. In Vitro Determination of UV Protective Effect of K. galanga Rhizome Oil and Microemulsion

The UV protective activity of *K. galanga* rhizome oil and microemulsion was evaluated in vitro using an ultraviolet transmittance analyzer, model UV-2000S. *K. galanga* rhizome oil and microemulsion were accurately weighed (0.0287 ± 0.005 g) and applied to the polymethyl methacrylate (PMMA) plates, which uniformly distributed over the entire surface using a glove-worn finger. UV transmission measurements from wavelengths of 290 to 400 nm were completed using a spectrophotometer equipped with an integrating sphere. The photoprotective effect of *K. galanga* rhizome oil and microemulsion was reported as sun protection factor (SPF) values. In this technique, calculation of SPF was automatically performed using the following equation (Equation (4)).

(4) $$SPF=\frac{\int \int_{290\;\mathrm{nm}}^{400\;\mathrm{nm}}E_{\lambda }\cdot S_{\lambda }d_{\lambda }}{\int \int_{290\;\mathrm{nm}}^{400\;\mathrm{nm}}E_{\lambda }\cdot S_{\lambda }\cdot T_{\lambda }d_{\lambda }}$$

where *E_λ_* is the relative erythemal action spectrum, *S_λ_* is the standard solar spectral irradiance, and *T_λ_* is the optical total transmittance of the textile specimen [58].

### 4.13. In Vitro Cytotoxicity of K. galanga Rhizome Oil and Microemulsion against RAW 264.7 Cells

The effect of *K. galanga* rhizome oil and microemulsion on the cytotoxicity of RAW 264.7, a macrophage cell line, was measured via MTT assay [59]. Briefly, RAW 264.7 cells were plated at a density of 1 × 10^4^ cells/well and incubated for 24 h. After incubation, RAW 264.7 cells were treated with 100 µL of *K. galanga* rhizome oil at concentrations of 0.25–783 mg/mL and microemulsion containing *K. galanga* rhizome oil at concentrations of 0.07–231 mg/mL. The blank microemulsion was tested at concentrations of 0.29–935 mg/mL for 24 h. The samples were removed and replaced with 100 µL of 0.5 mg/mL MTT solution and incubated for 3 h at 37 °C, 5% CO_2_. The formazan crystals were solubilized in 100 µL DMSO. The absorbance was determined at 550 nm using a multi-mode microplate reader (Spectra Max M3, Molecular Devices, San Jose, CA, USA) equipped with SoftMax^®^ Pro 7 software (Molecular Devices, San Jose, CA, USA). Cell viability was calculated using the following equation.

(5) $$Cell\;viability\;\left(\%\right)=\frac{A550\;sample}{A550\;control}\times 100\%$$

### 4.14. In Vitro Anti-Inflammatory Assay of K. galanga Rhizome Oil and Microemulsion 

The anti-inflammatory effect of *K. galanga* rhizome oil and microemulsion was determined via nitric oxide (NO) secretion from macrophage cells. Briefly, RAW 264.7 cells were plated at a density of 1 × 10^4^ cell/well in a 96-well plate. Cells were induced with 50 ng/mL LPS in the presence or absence *K. galanga* rhizome oil (0.03–3.92 mg/mL), *K. galanga* rhizome oil microemulsion containing oil of 0.005–0.58 mg/mL, and blank microemulsion (0.29–935 mg/mL) in different concentrations. The cells were incubated for 18 h. At the end of the incubation, an equal volume of medium was mixed with Griess reagent, which was made by combining 20 mg/mL sulfanilamide and 1 mg/mL N-(1-naphthylethylenediamine in 5% phosphoric acid in a 1:1 ratio. The absorbance of cell supernatant resulted from the reaction was read at 540 nm to quantify the nitrite levels using a UV-Vis spectrophotometer microplate reader (Spectra Max M3, San Jose, CA, USA) equipped with SoftMax^®^ Pro 7 software. The amount of nitrite was calculated from the sodium nitrite standard curve. The treated cells were then tested for cell viability using MTT assay. The medium was replaced with 0.5 mg/mL MTT reagent (100 µL/well) and incubated for 3 h. The formazan product was measured at 550 nm using microplate reader UV-Vis spectrophotometry (SpectraMax M3, San Jose, CA, USA).

### 4.15. Formulation of K. galanga Rhizome Oil Microemulsion-Based Hydrogel

The six formulations of hydrogel containing *K. galanga* rhizome oil microemulsion were prepared. The hydrogel base formulations were prepared from various concentrations of sodium carboxymethyl cellulose, i.e., 1% *w*/*w*, 1.5% *w*/*w*, 2% *w*/*w*, 3% *w*/*w*, 4% *w*/*w*, and 5% *w*/*w*. *K. galanga* rhizome oil microemulsion (85% *w*/*w*) was then slowly added to the hydrogel base (14% *w*/*w*) and mixed thoroughly. The concentration of microemulsion incorporated into the hydrogel was calculated based on its UV protective activity. Phenoxyethanol (0.5% *w*/*w*) and tocopherol acetate (0.5% *w*/*w*) were added as a preservative and antioxidant, respectively.

### 4.16. Appearance, Rheology, pH, and Physical Stability of K. galanga Rhizome Oil Microemulsion-Based Hydrogel

The appearance and homogeneity of the hydrogel formulations containing *K. galanga* rhizome oil microemulsion were visually observed. The best formulation was inspected to characterize for the pH, viscosity, rheology, and physical stability. The pH of the hydrogels was determined using a pH meter (Orion model 320, Thermo Electron Corporation, Waltham, MA, USA). The viscosity and rheology of hydrogels were measured using a Thermo Scientific HAAKE RheoStress 1 rheometer with a plate and plate geometry (Waltham, MA, USA) (1.0 mm gap, 60 mm diameter). The hydrogel’s temperature was set to 25 °C. The shear rate ranged from 0.5 to 100 s1 with a frequency of 1 Hz.

Heating-cooling cycles were used to conduct an accelerated stability study of *K. galanga* rhizome oil microemulsion-based hydrogel. The test was completed in 12 days (six cycles) [60]. In each cycle, the freshly prepared microemulsion-based hydrogel was stored at 4 °C for 24 h and 45 °C for another 24 h. For the accelerated stability study, the hydrogel was stored at 4, 30, and 45 °C for 90 days. After completing heating/cooling cycles and at days 0, 30, 60, and 90, hydrogels were observed for pH, viscosity, and rheology. 

### 4.17. Statistical Analyses

The statistical analysis was carried out using GraphPad Prism 7.0 (GraphPad Software, San Diego, CA, USA). The data are presented as mean SD. An analysis of variance was used to assess the statistical difference in size, PDI, and zeta potential values (one-way ANOVA). The significance of the differences was determined using Tukey’s post hoc test. A significance level of *p* < 0.05 was used in all cases.

## Figures and Tables

**Table 1:** The retention time and percentage chemical composition of the compounds identified by the GC-MS analysis of the essential oil distilled from the rhizome of *K. galanga*.

Retention Time (min)	% Area	Chemical Name	CAS NO.	Quality
4.354	0.11	α–Tricyclene	508-32-7	96
4.507	0.53	L-α-Pinene	7785-26-4	97
4.728	1.45	Camphene	79-92-5	97
5.024	0.25	3,7,7-Trimethyl-1,3,5-cycloheptatriene	3479-89-8	97
5.126	0.25	2(10)-Pinene	123-91-3	95
5.256	0.14	β-Myrcene	123-35-3	97
5.426	0.10	Octanal	124-13-0	87
5.589	3.79	3-Carene	13466-78-9	96
5.790	0.81	o-Cymene	527-84-4	97
5.854	0.50	D-Limonene	5989-27-5	99
5.910	6.19	Eucaliptol	470-82-6	98
7.637	0.09	Camphor	76-22-2	97
7.810	0.75	p-Mentha-1,5-dien-8-ol	1686-20-0	83
7.926	4.32	endo-Borneol	507-700-0	97
8.054	0.20	2-Caren-4-ol	6617-35-2	97
8.103	0.40	m-Cymen-8-ol	5208-37-7	95
8.165	0.24	p-Cymen-8-ol	1197-01-9	93
8.262	0.20	L-α-Terpineol	10482-56-1	91
8.739	0.15	Eucarvone	503-93-5	86
10.010	0.16	4,7,7-Trimethylbicyclo (4.1.0) hept-3-en-2-one	81800-50-2	99
11.035	0.19	Tetradecane	629-59-4	98
11.248	0.71	Cyperene	2387-78-2	98
11.348	0.12	α-Gurjunene	489-40-7	97
11.965	36.33	Ethyl cinnamate	103-36-6	98
12.079	0.22	1-Ethyl-2-methyl cyclododecane	22681-52-3	94
12.296	13.12	Pentadecane	629-62-9	98
12.643	0.40	γ-Cadinene	39029-41-9	99
12.728	0.36	δ-Cadinene	483-76-1	98
12.844	0.16	Cubebol	23445-02-5	95
13.213	0.27	Germacrene B	15423-57-1	99
14.403	0.89	Ethyl p-methoxycinnamate	1929-30-2	99
14.490	0.22	6(E),8(E)-Heptadecadiene	2000321-41-2	93
14.568	0.30	(Z)-3-Heptadecene	2000328-51-7	98
14.808	0.31	Heptadecane	629-78-7	98
15.486	23.77	(E)-Ethyl-p-methoxycinnamate	24393-56-4	99

## Data Availability

Not applicable.
